# Influence of Stress Field and Temperature Field on Residual Stress of 2A14 Aluminum Alloy Based on In Situ SAXS Method

**DOI:** 10.3390/ma16010170

**Published:** 2022-12-24

**Authors:** Guanghui Yang, Bing Xue, Zhengyi Li, Gang Zhou, Shaohua Zhang, Ning Lu, Lei Wen, Duzhou Zhang

**Affiliations:** 1 National Center for Materials Service Safety, School of Mechanical Engineering, University of Science and Technology Beijing, Beijing 100083, China; 2 Beijing Institute of Control Engineering, Beijing 100081, China; 3 China Academy of Space Technology, Beijing 100081, China

**Keywords:** synchrotron radiation, residual stress, aluminum alloy, thermo-mechanical coupling

## Abstract

In this paper, based on in situ synchrotron radiation SAXS technology, the effects of stress field, temperature field and thermo-mechanical coupling conditions on the evolution of residual stress are discussed, respectively. The results show that the continuous increase of the external load led to the increase of the residual stress perpendicular to the tensile direction of the 2A14 aluminum alloy, and when the external load closed to the yield strength, the change of the residual stress was no longer significant. Under the action of temperature, the residual stress of 2A14 aluminum alloy reduced after the process of heating–holding–cooling. Under the action of thermo-mechanical coupling, the recovery effect of aluminum alloy was triggered, the energy storage of deformation was released, the lattice strain was reduced and the residual stress introduced by external load was reduced.

## 1. Introduction

The 2000 series aluminum alloys are widely used in aerospace equipment, such as control moment gyroscopes (CMG), due to their excellent mechanical properties. The mechanical properties have been effectively improved with strengthening heat treatment and processing [1], but a high level of residual stress is introduced [2], especially when the material is significantly thick, creating a high probability of internal cracking, which can damage the material’s serviceability seriously [3,4,5,6]. The distribution of residual stresses can seriously affect the dimensional stability and service indexes, such as fatigue strength, hardness and corrosion resistance of the parts, so detailed residual stress characterization of materials is required [7,8,9]. The main methods of eliminating residual stresses in aluminum alloys are prestretching and heat treatment. If the artificial aging temperature is too high, the aging temperature is too low (<140 °C), and it will decrease the strength of the material, resulting in only 10–43% of the residual stress being eliminated [10]. Robinson studied the effect of quenching speed on the properties of 7050 aluminum alloy [11]. The results showed that the residual stress was reduced by up to 78% and the mechanical properties only decreased by 9% when boiling water was used as the quenching medium. Ferreira-Barragans found that residual stress reduction became insignificant after the aging time increased beyond 10 h [12]. Uphill quenching can reduce residual stress by about 30% [13,14]. Studies by Robinson and Prime showed [15,16,17,18] that the application of 2–3% preloading for sheet structure reduced residual stress significantly.

The residual stress is measured by drilling and layer-by-layer methods, and although they have high measurement accuracy, they belong to the category of destructive tests. Residual stress’ nondestructive testing methods such as X-ray diffraction (XRD) and ultrasonography have appeared in recent years, especially the synchrotron radiation X-ray diffraction technology, which measures the residual stress according to the change of the interplanar spacing and realizes the rapid determination of the spatial residual stress state inside the material [19]. The technology and theory of X-ray diffraction are relatively mature. Synchrotron X-ray diffraction technology in this technology has better effective penetration depth and more accurate measurement results than ordinary X-ray diffraction due to the advantages of high flux, high brightness and small divergence over conventional X-rays [20,21,22]. Koro used synchrotron radiation X-ray diffraction technology to conduct nondestructive testing of the residual stress tensor in Cu-TSV and believed that synchrotron radiation is an effective measurement tool [23]. Webster used synchrotron radiation to measure the residual stress distribution on the cross-section of the friction stir welding of AA7108 aluminum alloy, and the results showed that the method could nondestructively measure the surface residual stress [24]. Beaudoin measured the change of residual stress of Al alloy during an in situ tensile process using synchrotron radiation technology; the study shows that the stress states of grains in different areas are different during the tensile process, and the yield phenomenon tends to occur in the preferred orientation grain region [25,26]. According to the current public literature, in situ studies of residual stress of aluminum alloy by synchrotron radiation technology are still few.

This paper mainly studies the evolution of residual stress of 2A14 aluminum alloy under external load, temperature and its coupling. The effects of stress field, temperature field and thermo-mechanical coupling on the evolution of residual stress in aluminum alloys are discussed by using the synchrotron radiation in situ characterization technique.

## 2. Materials and Methods

### 2.1. Materials

**Figure 1:**
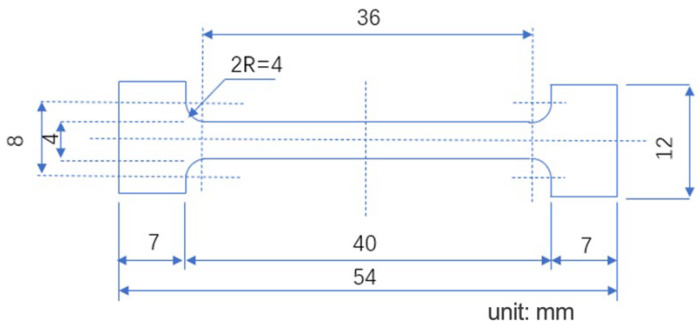
Tensile specimen size.

2A14 aluminum alloy along the rolling direction was used in this experiment. 2A14 tensile specimen sizes are shown in Figure 1. The initial heat treatment state of the specimen was the state of solid solution aging. The 40 mm was used for the in situ measurement of synchrotron radiation. The chemical composition of 2A14 aluminum alloy is shown in Table 1. All the specimens were treated with a solid solution at 500 °C for 2 h, and the quenching medium was 20 °C water, according to the standard YS/T591-2017.

### 2.2. Characterization of Residual Stress and Experiment Methods

#### 2.2.1. Synchrotron Radiation X-ray Diffraction

**Figure 2:**
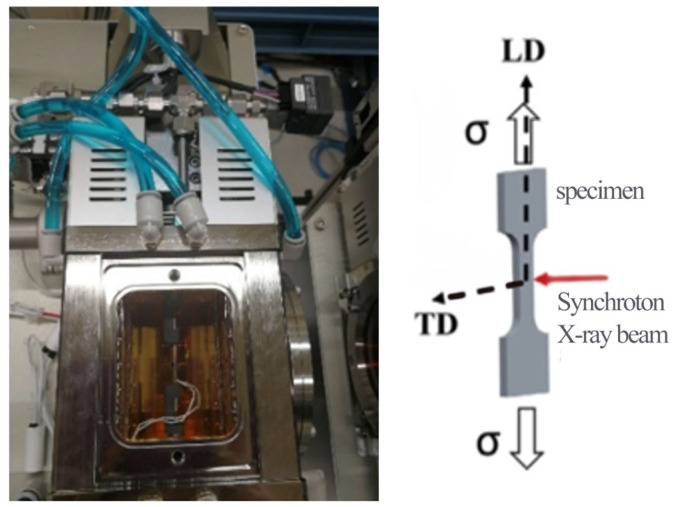
Synchrotron radiation in situ stretching device.

We have proven in previous studies that the residual stress testing method of synchrotron radiation is highly consistent with the laboratory XRD method of residual stress testing, so in this paper, synchrotron radiation technology is used to measure residual stress changes. The experiment of X-ray transmission diffraction based on in situ synchrotron radiation was conducted at the BL08B2-SAXS station of Spring-8 in Japan. The synchrotron radiation in situ stretching device is shown in Figure 2. In the experiment, the incident light spot size was 0.12 mm × 0.45 mm, the energy of the rays was 30 KeV, and the wavelength was 0.0413217 nm. The in situ loading experiments were carried out in transmission mode, and the 2A14 aluminum alloy tensile sample was perpendicular to the incident light direction and fixed to the holding end. When the incident light hit the sample in transmission mode, the diffraction information was collected by a detector installed 205 mm away from the sample. CeO_2_ was the standard calibration parameter. The collected two-dimensional diffraction information was processed by Fit 2D software (v18.002) to obtain a one-dimensional diffraction pattern. The stress measurement of synchrotron radiation used in this experiment was based on sin^2^ ψ, and the details can be found in previous research [27].

#### 2.2.2. Load Stress

Static loads were applied step by step during the tensile process; the stress rate and range were 40 N/min and 0–400 MPa, respectively. When stress was loaded stably for 1 min, the diffraction information of Al(311) crystal plane was collected by a two-dimensional surface detector. After completing collection, the next stress level was acquired, and the variation of Al(311) plane diffraction peak was measured during the whole stress loading process. All experiments tested three parallel samples.

#### 2.2.3. Temperature Test

The 2A14 aluminum alloy samples were heating–holding–cooling, and the heating rate was 13–15 °C/min. During the heating and cooling process, the diffraction information was collected every 5 min. In the temperature field, the heating temperature was 200 °C, and the holding time was 30 min. When the temperature reached 200 °C, it was held for 10 min, 20 min and 30 min, respectively, and then we collected the diffraction information. In thermo-mechanical coupling, the holding heating temperature was 200 °C and 280 °C, respectively. The holding time was for 60 min.

## 3. Results and Discussion

### 3.1. Diffraction Information of 2A14 Aluminum Alloy under Stress Field

**Figure 3:**
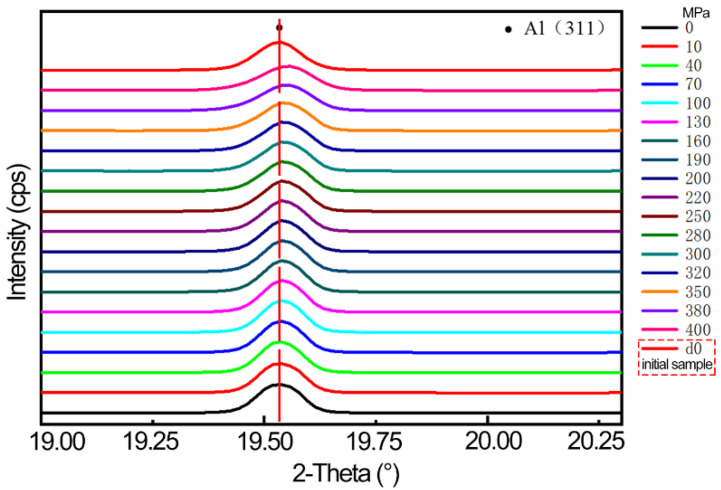
Diffraction integral maps under different loads.

In situ tensile synchrotron radiation experiments were carried out on 2A14 aluminum alloy tensile samples at the BL08B2-SAXS station of Spring-8 in Japan. The one-dimensional integral diffraction peak shape and evolution law of different stress levels measured in the experiment are shown in Figure 3. In the process of in situ experiments, the crystal plane spacing of the initial state of the sample is d_0_. The diffraction angle and half-height width of Al(311) crystal plane gradually change, indicating that the grain deforms under the action of external load during the tensile process, which led to the change of crystal plane spacing.

**Figure 4:**
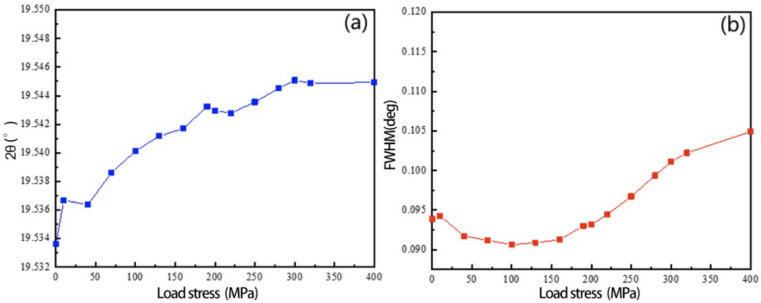
Variation of Al(311) during in situ loading process. (**a**) Diffraction angle; (**b**) FWHM.

To observe more clearly the evolution characteristics of the residual stress of the 2A14 aluminum alloy sample during the loading process, the Al(311) diffraction peak at the test position was analyzed in detail. Figure 4a shows the change of the diffraction angle of the Al(311) peak in the process of external loading. The 2θ increases to 19.545° with the increase of the external loading, indicating that the Al(311) peak moves toward the direction of the higher diffraction vector during the process of external loading. Figure 4b shows the changing trend of the full width at half maximum (FWHM) in the process of external loading. The FWHM increases from 0.094° initially to 0.105° at 400 MPa, indicating that the grain size perpendicular to the tensile direction decreases during the tensile process. Because the load is applied, the 2A14 aluminum alloy deforms by an external force; the sample is stretched longitudinally and contracted transversely; the grains in the measurement direction undergo elastic compression deformation; the interplanar spacing decreases; the diffraction angle increases; and the mutual extrusion between grains in the process of load application make the grain size slightly decrease and the half-height width increase.

**Figure 5:**
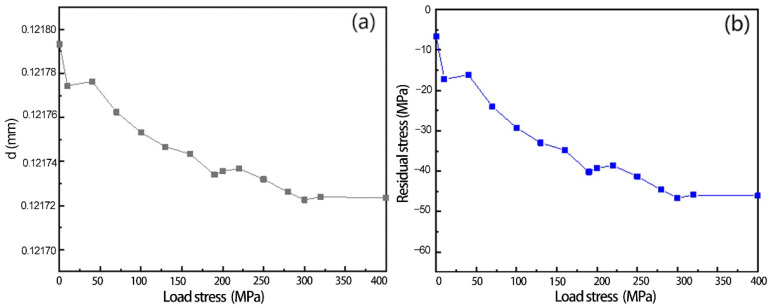
Variation of in situ loading process. (**a**) Crystal plane spacing; (**b**) residual stress.

Based on the diffraction angle obtained after fitting and the Bragg equation [28], the crystal plane spacing and residual stress changes were calculated, the results of which are shown in Figure 5. The initial residual stress of the 2A14 aluminum alloy tensile sample is −6.58 MPa, and the residual stress level increases gradually with the gradual application of the load. The rate of increase of residual stress is maxed when 10 MPa is applied at the beginning and gradually stabilizes at 300–400 MPa. The reason is that the grain in the test direction is the elastic stage at the beginning, the compressive deformation is relatively large, the crystal plane spacing decreases rapidly, and the residual stress level increases. When the load increases to about 300 MPa, which is close to the tensile strength of 2A14 aluminum alloy, the increase of external load does not cause large deformation of 2A14, and the change of residual stress gradually becomes stable.

### 3.2. Diffraction Information of 2A14 Aluminum Alloy under Temperature Field

**Figure 6:**
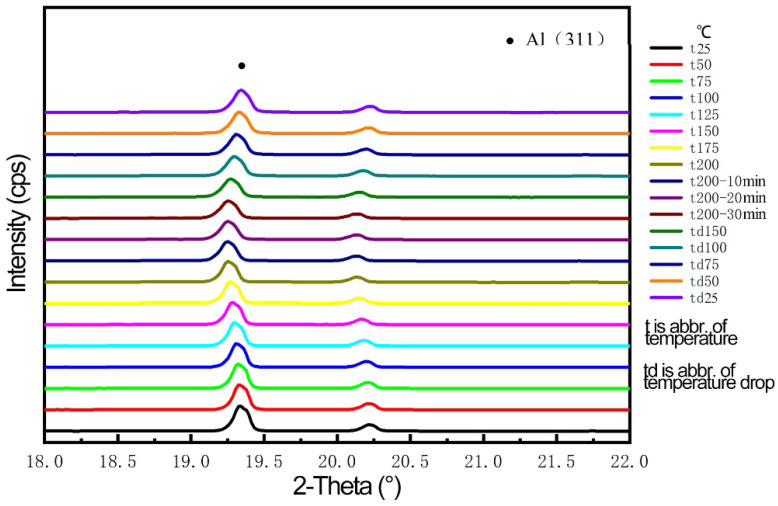
Variation of diffraction angle during different temperatures.

As the 2A14 aluminum alloy samples underwent heating–holding–cooling, the temperature was set at 200 °C for 30 min, and the heating rate was 13–15 °C/min. When the temperature reached 200 °C, it was held for 10 min, 20 min and 30 min, respectively, and then we collected the diffraction information. During the heating and cooling process, the diffraction information was collected every 5 min. The diffraction information with different temperatures is shown in Figure 6.

**Figure 7:**
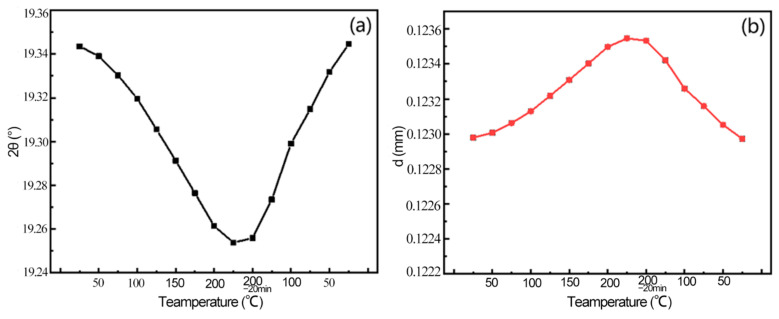
Variation in the process of temperature–insulation–cooling. (**a**) Diffraction angle; (**b**) crystal plane spacing.

Figure 7 shows the change of diffraction angle and crystal plane spacing in the process of heating–holding–cooling. Figure 7a shows the change of the Al(311) peak diffraction angle in the process of heating–holding–cooling: the Al(311) diffraction angle moves toward the low diffraction direction with the temperature increases and gains stability at 200 °C, and the lowest diffraction angle is 19.2559° after holding for 20 min; then the diffraction angle begins to shift to a high angle and finally reaches 19.3445° in the cooling process. Figure 7b shows the change of the interplanar spacing of Al(311) during the heating–holding–cooling process: When the temperature increases to 200 °C, the interplanar spacing increases from 0.12306 nm at the beginning to 0.12355 nm. In the holding stage, the crystal plane spacing gradually decreases to 0.12342 nm after holding 20 min. In the cooling stage, the crystal plane spacing decreases with the decrease of temperature, and the crystal plane spacing decreases to 0.12297 nm when the temperature reduces to room temperature.

**Figure 8:**
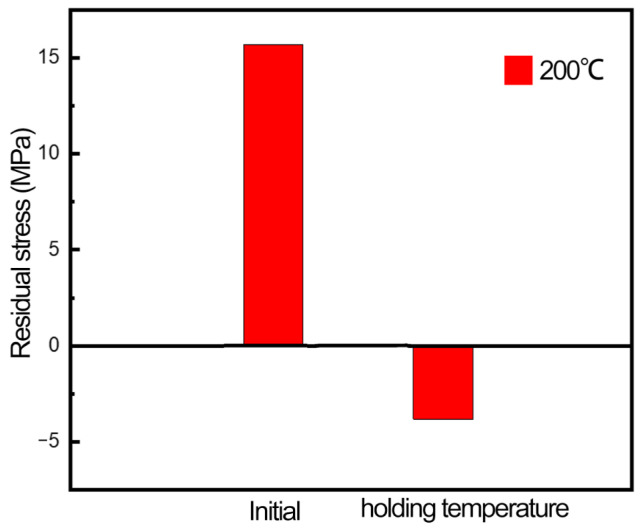
Residual stress comparison between holding temperature and initial state.

Figure 8 shows the comparison of residual stress before and after the heating–holding–cooling process. In the heating stage, the crystal plane spacing gradually increases, and the diffraction angle decreases with the increase of temperature, indicating that temperature contributes to the growth of grains. When the temperature rises to 200 °C, the sample does not have any deformation energy storage due to the fact that it has not been loaded before. Thus, it is difficult to produce the recovery phenomenon, and the diffraction angle and crystal plane spacing are almost unchanged. In the cooling stage, the crystal plane spacing gradually decreases, and the diffraction angle gradually increases with the decrease of temperature. After the process of heating–holding–cooling at 200 °C, the grains are thermally expanded firstly, then dislocation movement occurs at high temperature, and the lattice distortion decreases. When the temperature cools to room temperature, the grains undergo expansion and contraction, parts of the strain between grains are released and the distance between crystal planes and the residual stress level are reduced.

### 3.3. Diffraction Information of 2A14 Aluminum Alloy under the Alternating Action of Thermo-Mechanical Coupling

The samples were subjected to static tensile gradually and held at temperature after unloading the external static load. The heating temperature was 200 °C and 280 °C, respectively, and the heating rate was 13–15 °C/min. The target temperature of group A was 200 °C. After gradually heating to the target temperature and kept at 200 °C for 60 min, they were then slowly lowered to room temperature (below 30 °C). The target temperature of group B was 280 °C. Firstly, the step-by-step static load test was carried out and then unloaded. The holding time at the target temperature was the same as group A, and then the temperature was lowered slowly to room temperature. The diffraction information was collected every 5 min throughout the whole process of heating–holding–cooling, and the diffraction information with temperature change was measured.

**Figure 9:**
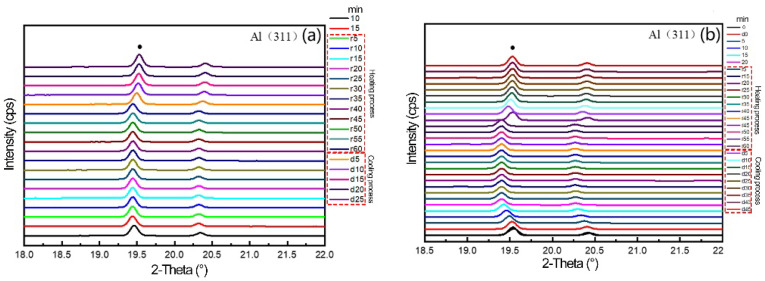
Diffraction pattern of temperature change after in situ loading: (**a**) 200 °C, (**b**) 280 °C.

The diffraction peak and evolution law of the samples collected in the experiment are shown in Figure 9. Figure 9a,b show the variation of the diffraction angle of Al(311) peak in the process of heating–holding–cooling at the target temperature of 200 °C and 280 °C after load unloading.

**Figure 10:**
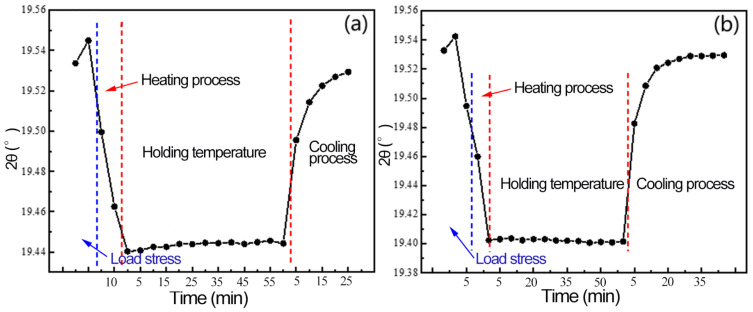
Diffraction angle changes under multiple fields: (**a**) 200 °C, (**b**) 280 °C.

The diffraction changes of Al(311) peak of 2A14 aluminum alloy in the process of temperature change were analyzed in detail. Figure 10a shows the changes of the diffraction angle of Al(311) peak in the process of heating–holding–cooling at 200 °C after load unloading. Figure 10b shows the change of 280 °C process. The 2θ of the two groups of samples moves toward the direction of the low diffraction vector during the heating process, but there is no obvious shift in the holding process, and the diffraction angle moves toward the direction of high diffraction vector in the cooling process. When the external force is unloaded, the two groups of samples enter the heating stage, and the diffraction angle moves toward the direction of the low diffraction vector. At this time, the 2θ angle of group A samples moves slightly toward the direction of high diffraction, but the change is not obvious and stabilizes around 19.445°. Finally, the 2θ angle of group B does not shift significantly and remains at around 19.400°. After cooling to room temperature, the diffraction angle of group A is 19.529°, and that of group B is 19.53°; there is not much difference between them.

**Figure 11:**
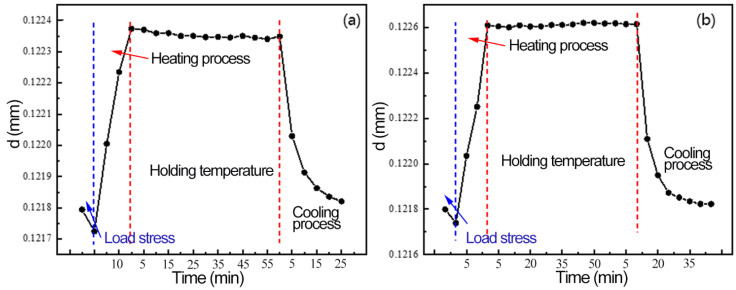
Variation of crystal plane spacing under multiple fields: (**a**) 200 °C, (**b**) 280 °C.

Figure 11 shows the change of Al(311) interplanar spacing during the heating–holding–cooling process. After the external load is removed, a large amount of plastic deformation occurs in the sample, the grain in the measured direction is compressed and the diffraction angle moves correspondingly to the high diffraction angle. During the heating process, the aluminum alloy expands with the increase of temperature, and the interplanar spacing increases. In the holding process, aluminum alloy produces a recovery phenomenon firstly, which consumes a part of the strain energy stored by plastic deformation in the tensile process; reduces the lattice distortion; releases a part of residual stress; and reduces the crystal plane spacing slightly. Finally, the crystal plane spacing of groups A and B are 0.121821 nm and 0.12182 nm, both of which are lower than that of the two groups of samples at the initial state.

**Figure 12:**
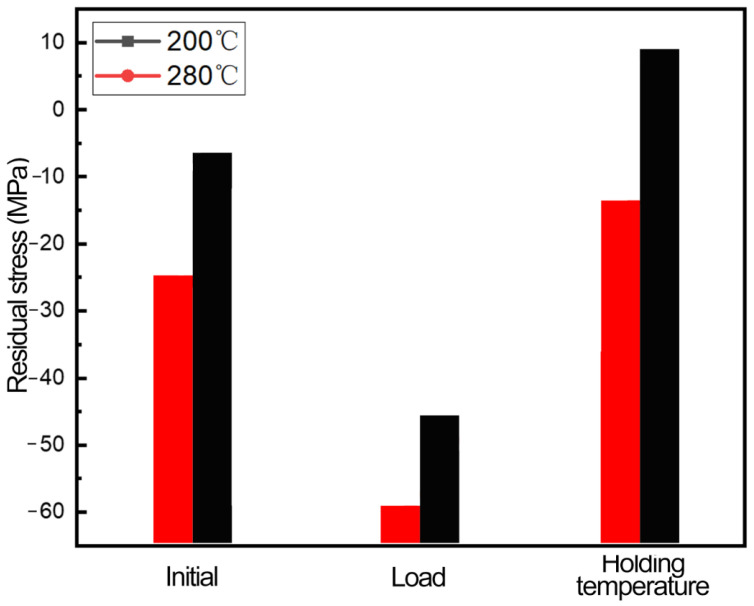
Stress variation at different stages of the thermo-mechanical coupling process.

Figure 12 shows the variation of residual stress after the initial state, loading state and heating–holding–cooling of the sample. The initial stress state of the sample is not high, and the residual stress increases significantly in the direction of the test due to the mutual tensile between the grains. When the aluminum alloy undergoes heating–holding–cooling at different temperatures, samples experience the recovery phenomenon and release a small part of the deformation energy storage, so the residual stress is reduced to a lower level than that at the initial state. The residual stress level of the samples in groups A and B is 18.85% and 45.69%, respectively, indicating that the reduction of residual stress is significantly improved by increasing the temperature.

## 4. Conclusions

The effects of stress field, temperature field and thermo-mechanical coupling conditions on the evolution of residual stress were discussed and analyzed by using synchrotron radiation in situ characterization technology. The main findings were as follows:

(1) With the continuous increase of the external load, the residual stress of the 2A14 aluminum alloy perpendicular to the tensile direction gradually increases. When the external load is greater than 320 MPa (Yield strength of 2A14 aluminum alloy), the residual stress changes are no longer significant.
(2) Under the action of the temperature, the grains experience expansion, recovery and shrinkage phenomena when 2A14 aluminum alloy undergoes a heating–holding–cooling process; the grain deformation releases a small part of the lattice distortion and reduces a part of the residual stress.
(3) Under the action of thermo-mechanical coupling, the 2A14 aluminum alloy triggers the recovery effect, releases the deformation energy storage, reduces the lattice strain and reduces the residual stress introduced by the external load in the heating–holding–cooling process; moreover, the stress reduction effect at 280 °C is better than that at 200 °C.

## Figures and Tables

**Table 1:** Chemical composition of 2A14 aluminum alloy (wt.%).

Cu	Mg	Mn	Si	Fe	Zn	Al
4.32	0.64	0.84	0.85	0.29	0.08	Bal.

## Data Availability

The raw/processed data required to reproduce these findings cannot be shared at this time as the data also form part of an ongoing study.
